# Chemical alterations of grain surface by cold plasma technology: Comparison of buckwheat and wheat grain responses to oxygen low-pressure plasma

**DOI:** 10.1016/j.heliyon.2023.e20215

**Published:** 2023-09-15

**Authors:** Pia Starič, Aleš Kolmanič, Ita Junkar, Katarina Vogel-Mikuš

**Affiliations:** a Biotechnical Faculty, University of Ljubljana, Jamnikarjeva ulica 101, Ljubljana, Slovenia; b Agricultural Institute of Slovenia, Hacquetova ulica 17, Ljubljana, Slovenia; c Institute Jožef Stefan, Jamova cesta 39, Ljubljana, Slovenia

**Keywords:** ATR-FTIR, Cold plasma, Germination, MDA, Phenols, Seed

## Abstract

Cold plasma (CP) has a great potential for decontamination or improvement of grain germination. However, disputing results have been reported, as plasma treatment can affect species and varieties of grains in different ways. The differences may be due to the chemical composition of grain pericarps, the structure of the grains and metabolic response mechanisms. CP treatment decreased grain germination rate, speed and activity of α-amylase of buckwheat grains. Such effects on both varieties of wheat grains were present after longer exposure to plasma. Lipid peroxidation was highest in buckwheat grains, whereas wheat grains were less affected. Plasma-treated Gorolka variety exhibited a low level of lipid peroxidation, no different to untreated grains, compared to Primorka grains, where longer treatment triggered higher levels of lipid peroxidation. The response of grains to CP treatment depends on the chemical and structural properties of grains pericarp, as well as plant tolerance to certain abiotic conditions.

## 1 Introduction

In recent years, cold plasma (CP) treatment of grain and other agricultural products is gaining increasing interest. CP is an ionized gas that causes chemical and/or morphological changes on the surface of grains. In addition to a decontamination effect on the seed surface, CP can also influence plant metabolism, resulting in improved germination, growth, or even increased yield and stress resistance [1].

Plasma interactions with grains occur primarily on the grain surface, where the different chemical compositions of various seeds and grains may play an important role in the response mechanisms to CP treatment [2,3]. Surface changes include functionalization of the grain surface, mainly oxidation if oxygen plasma is used, and/or changes in grain morphology [[4], [5], [6], [7]]. The result of the changed surface chemistry of grains is an increase in hydrophilic properties of the pericarp and improved wettability and water uptake of grains [7,8]. Surface changes are sometimes accompanied by decontamination effects and alterations in plant metabolism, germination, growth and development [1,9,10]. Effects of CP treatment on grain metabolism can be easily seen in changed germination characteristics such as speed of germination and final germination rate. In correlation to the germination patterns, the activity of α-amylase enzyme is usually altered, as it is an important factor in grain germination. It enables the degradation of starchy endosperm, a nutritional reserve that is made available for the grain embryo to use for its growth and development upon imbibition [[11], [12], [13]]. CP discharge contains several components which can act as stressors for the grain. One such stressor could be the loss of moisture during the treatment, and another is the reactive oxygen species (ROS) present in the discharge [14]. The stressors can cause the oxidation of cell membranes (lipid peroxidation). The by-product of this process is malondialdehyde (MDA) which can be measured and quantified spectrophotometrically [[15], [16], [17]].

Many researchers in the field of plasma agriculture agree that the effects of CP treatment on grain depend on the type of plasma and gas used. The properties of plasma discharge can vary from one plasma reactor to another and even from moisture level in the reactor in gas or/and from grains [1,18]. It is agreed that the response to plasma treatment by grains/plants is not uniform; it is more or less species and even variety-dependent [15,19,20].

Cereals represent a large part of human nutrition, with wheat being the most prominent. However, pseudo cereals, such as common buckwheat, are once again gaining interest in the human diet because of their excellent nutrient profile. By antioxidant composition, buckwheat has higher antioxidant activity compared to wheat and is rich in high-quality proteins, dietary fibres and lipids (polyunsaturated fatty acids) [21]. Besides differences in nutrient composition, there are physiological and structural variations between common buckwheat (*Fagopyrum esculentum* Monech) and winter wheat (*Triticum aestivum*). Wheat is a monocot and is a member of a large family of grasses. Its grain is built in several layers: bran (outer pericarp, inner pericarp, testa, hyaline layer), aleurone layer and endosperm. Bran can be up to 65 μm thick and protects the embryo from abiotic stresses such as UV radiation, drought and others [22].

Buckwheat is a dicotyledonous plant classified as a pseudo-cereal because of its similarities with cereals. Buckwheat endosperm is similar to cereals and grain; it also contains an aleurone layer between the endosperm and grain pericarp. Buckwheat pericarp thickness is similar to wheat bran. However, the similarities between the two crops end here. The pericarp of buckwheat grains consists of epicarp, pigment layer and endocarp followed by testa, which consists of epi- and endo-carp [23].

Besides the structural differences in the composition of grain coats, chemical differences are also present. The chemical composition of each species and variety may, to some extent, direct the interaction of plasma with the grain surface. The chemical and structural composition of the pericarp is important for grain germination and development in the first stages of growth. Pericarp is an important barrier between grain and its environment. It acts as a protection against biotic and abiotic stress and regulates water absorption and grain dormancy/germination [[24], [25], [26], [27]]. Thus, chemical changes on grain surface may contribute to specific species/variety response and sensitivity to CP treatment. When treating two different mutants and one wild-type batch of *Arabidopsis thaliana* seeds, germination of mutant *gpat5* was negatively affected by CP treatment, and even untreated seeds had a lower germination rate compared to wild-type seeds. The *gpat5* mutant has, among other characteristics, a lower concentration of suberin in the seed coat. Thus, the results discussed in the manuscript could also indicate the importance of the chemical composition of seeds/grains in their response to CP treatment [6].

The objective of this study was to assess the impact of CP treatment on various species and varieties of grains. The metabolic effects of CP treatment were determined through germination tests and α-amylase activity measurements. To evaluate if the CP treatment induced oxidative stress, the MDA content of the grains was measured. The chemical structure of the grains was also investigated through the assessment of total phenol content and FTIR analysis of the pericarps, as plasma is known to induce changes in chemical composition.

## 2 Methods

### 2.1 Grain material and cold plasma treatment

Grain of common buckwheat ‘Trdinova’ (*Fagopyrum esculentum* Trdinova) from the 2020 harvest year was obtained from a local farm (Rangus mlinarstvo in trgovina, Dol. Vrhpolje d.o.o., Dolenje Vrhpolje 15, 8310 Šentjernej, Slovenia). The grains of two winter wheat varieties, Gorolka and Primorka (*Triticum aestivum* L.) were obtained from Agricultural institute of Slovenia. The grains originated from the multiplication of varieties during the 2019/2020 growing season at their Infrastructure Center in Jablje (Mengeš, Central Slovenia). The grains were stored at 4 °C in the dark from harvest to the start of the experiments.

Grains were treated in a small radio-frequency (RF) inductively coupled plasma system. The RF generator was operating at 13.56 MHz frequency and 200 W power at 50 Pa pressure. Gas supplied to the chamber was oxygen with 99.999% purity [28]. The grains were placed on an aluminium tray inside the reactor glass tube and treated with plasma for 0, 5, 30 and 60 s.

### 2.2 Germination

Germination tests were performed 24 h after plasma treatment in Petri dishes (φ = 7 cm; 50 grains per Petri dish) lined with two layers of filter paper soaked in dH_2_O and kept in the dark in controlled conditions with day: night cycle 22 °C and 19 °C with four technical replicates per treatment. The number of germinated grains was counted on days 1, 2, 3, 7 and 10 from the imbibition. The final germination rate (G) on day 10 was calculated using the formula:

$$G=\frac{N_{g}\times 100\;\%}{N_{t}}$$

where N_t_ represents the total number of grains in a Petri dish, and N_g_ represents the number of germinated grains. The mean germination rate (MR) was calculated using the following formula:

$$\text{MR}=\frac{1}{\frac{\sum n\times d}{G_{T}}}$$

where n represents the number of germinated grains on day d, G_t_ represents the total number of germinated grains in a Petri dish.

### 2.3 α-amylase

The activity of α-amylase in the grain was measured following the user's manual of CERALPHA kit (Megazyme, Wicklow, Ireland). After soaking the grains for 16 h in distilled water, the grains were blotted with paper towels, frozen in liquid nitrogen and put in a freeze-dryer (Martin Christ Gefriertrocknungsanlagen, Osterode, Germany) at −30 °C and 0.340 mbar for 3 days. Dried grains were finely ground with a pestle in a mortar and α-amylase was extracted from 250 mg of homogenised material in a glass eprouvette using 5 mL extraction buffer solution (pH 5.4). After thoroughly mixing the plant material with the extraction buffer (vortexing), the samples were incubated in a water bath for 20 min at 40 °C and centrifuged at 1000×*g* for 10 min. For each sample, 100 μL of amylase reagent was transferred into 2 mL micro-centrifuge and pre-incubated in a water bath for 5 min at 40 °C. A volume of 100 μL of the sample was added to the amylase reagent, stirred and incubated in a water bath for further 10 min at 40 °C. After the incubation, 1.5 mL of stopping reagent was added and the solution was vortexed. The samples were centrifuged at 1000×*g* for 10 min. The absorbance of the samples was read at 400 nm (A_400_). α-amylase activity (CU g^−1^) was calculated using the following formula:

$$\alpha \;\text{amylase}\;\text{activity}=\frac{A400}{\text{incubation}\;\text{time}}\times \frac{\text{total}\;\text{volume}\;\text{in}\;\text{cell}}{\text{volume}\;\text{of}\;\text{extract}}\times 0.05525\times \frac{\text{extraction}\;\text{volume}}{\text{sample}\;\text{weight}}\times \text{dilution}$$

The result obtained in the calculation was multiplied by 4.1 to obtain values in international units of starch substrate (IU).

### 2.4 Light microscopy and sample preparation

Wheat grains were placed in a Petri dish layered with filter paper and wetted with dH_2_O. Grains were imbibed for 4 h and cut in half with a stainless-steel razor blade, embedded into tissue freezing medium (Leica Biosystems, Illinois, United States), mounted onto a metal holder and frozen in liquid nitrogen. Grain cross-sections were cut in cryomicrotome chamber (Leica CM3050, Nussloch, Germany) to 35 μm thick sections at −24 °C. The sections were put onto pre-cooled filter paper in pre-cooled aluminium beakers with covers. Samples were transferred to liquid nitrogen and freeze-dryed as described previously [29]. Dried sections were put on object glass, and a drop of dH_2_O water was added. The sample was then covered with a cover glass and observed and photographed under Axioskop 2 MOT microscope equipped with Axiocam MRc colour digital camera (Carl Zeiss Vision, Halbergmoos, Germany) using AxioVision 3.1 software. For each wheat variety, cross-sections of 7 grains were examined.

Photographs were analyzed in ImageJ software, where thickness of pericarp, testa, hyaline layer, total grain coat and aleurone layer was measured.

### 2.5 Lipid peroxidation and MDA content

The untreated and treated grains (50 grains) were frozen with liquid nitrogen and ground into a fine powder with a pestle and mortar. A sample of 80 mg of powder was weighed into 2 ml microcentrifuge tubes. For the extraction, 2 ml of extraction buffer EtOH: water was added in 80:20 ratio and mixed thoroughly. The samples were centrifuged at 3000 RCF for 10 min at 4 °C. The samples were held on ice the whole time.

In a new microcentrifuge tube, 800 μl of TBA buffer (20% (w/v) trichloroacetic acid, 0.01% butylated hydroxytoluene, 0.65% thiobarbituric acid) was added to 800 μl of the extracted sample and mixed thoroughly. The samples were incubated in an oven at 95 °C for 25 min. After incubation, the samples were put on ice and centrifuged at 3000 RCF for 10 min. Absorbance was measured against blank samples at 440, 532 and 600 nm. The MDA equivalent was then calculated by the following formulas:

$$MDA\;equivalent\left[\frac{nmol}{ml}\right]=\left(\frac{\left(A_{532}-A_{600}\right)-\left(\left(A_{440}-A_{600}\right)\cdot 0,0571\right)}{157000}\right)\cdot 10^{6}$$

$$MDA\;equivalent\;\left[\frac{nmol}{g}\right]=\frac{MDA\;equivalent\;\left[\frac{nmol}{ml}\right]*V_{sample}}{m}$$

Where V_sample_ is the volume of the sample and m is the dry weight of the sample used for the extraction.

### 2.6 Measurement of total phenolic content

The untreated or CP-treated grains were ground with millet for 25 s and put through a sieving shaker (Retsch, Germany). The pericarps of common buckwheat grains were collected on a 2 mm sieve, and the pericarps of both wheat samples were collected at 1 mm sieve. The samples were then ground to fine powder by a mixer mill (Star-Beater, VWR, Austria).

For each treatment, 200 mg of the material was weighed into the tubes in three replicates. A volume of 10 ml of 60% EtOH was added to the samples and mixed vigorously. The samples were left on a shaker overnight at room temperature. After incubation, the samples were centrifuged for 10 min at 2000×*g,* and the supernatant was stored in 15 ml Falcon tubes at 4 °C until use.

Total phenol content was measured by Folin-Ciocalteu reagent method. A volume of 50 μl of the extract was transferred to microcentrifuge tubes and mixed with 375 μl of dH_2_O and 25 μl of Folin-Ciocalteu reagent. Blank samples were prepared by mixing 50 μl of the extract with 400 μl dH_2_O. The samples (blanks as well) were incubated for 3 min at room temperature and 50 μl of 20% Na_2_CO_3_ was added. The samples were vortexed and incubated for 60 min at room temperature in the dark. The absorbance of the samples was measured at 750 nm with TECAN Infinite M plex microplate reader (Männedorf, Switzerland) on 96-well Brand microplates (transparent, flat bottom) with a measurement volume 200 ml. As a standard, different concentrations of catechol solutions were used.

### 2.7 Fourier transform infrared (FTIR) analysis

For the attenuated total reflection (ATR) Fourier transform infrared analysis (Alpha II, Bruker), the pericarps were removed from the grains after CP treatment with a scalpel and tweezers. The 2 × 2 mm large pericarp sections were put on the ATR diamond crystal so that the CP-treated site was in direct contact with the crystal, covering it entirely. The FTIR spectra were measured in 4–5 pericarp sections per treatment. The spectra were acquired at room temperature, in the 4000–800 cm^−1^ region, after 64 superimposed scans at 4 cm^−1^ spectral resolution.

### 2.8 Statistical analysis

The results of the study were presented as a mean ± standard error (SE) of two biological repeats. Statistical significance between the groups of samples was determined using one- or two-way analysis of variance (ANOVA) with Holm-Sidak post-hoc test at *p* value < 0.05. Statistical analysis and graphical visualization were carried out in SigmaPlot 12.0 software (Systat Software, San Jose, CA, USA). Cluster analysis on the basis of Euclidian distances and Ward agglomeration method was performed by XLSTAT (Lumivero) plug-in.

## 3 Results and DISCUSSION

### 3.1 Germination parameters and α-amylase activity of buckwheat and wheat grains

**Fig. 1:**
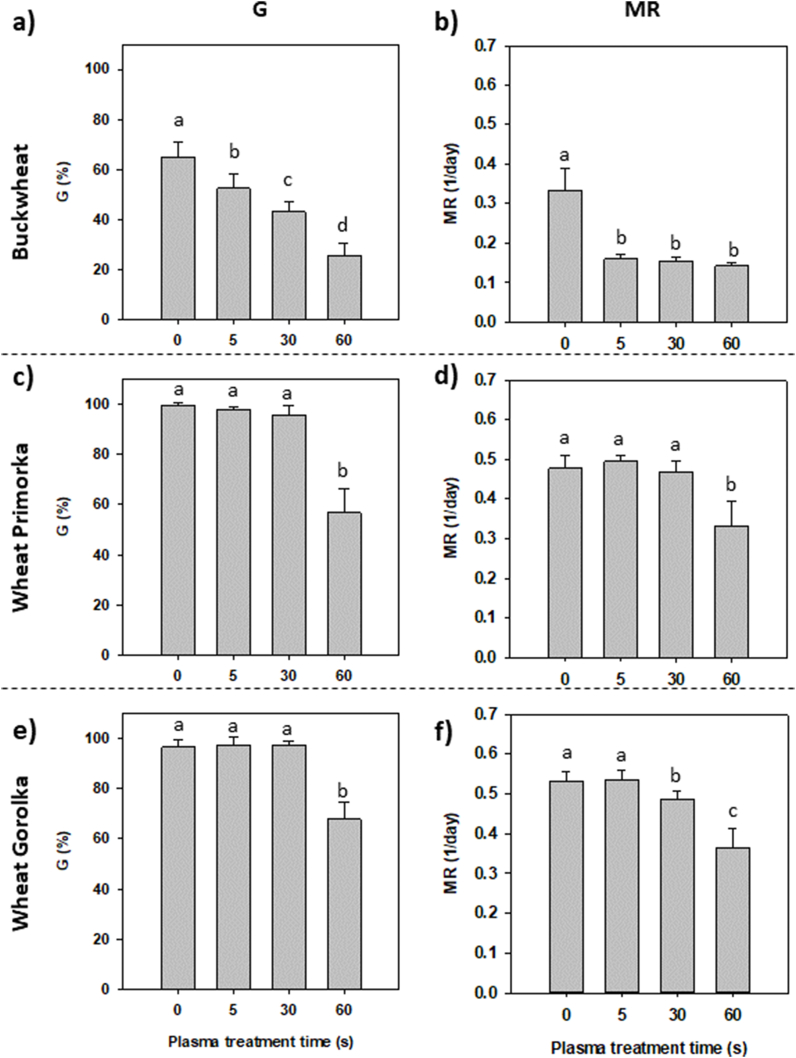
(a, c, e) Final germination rate G and (b, d, f) mean germination rate MR, of untreated (0 s) grains and CP-treated grains for 5, 30 or 60 s for buckwheat grains (a, b) and grains of wheat Primorka (c, d) and Gorolka variety (e, f). Displayed values are the mean ± SE of three replications. Different letters (a–c) indicate statistically significant differences among treatments according to Holm-Sidak post-hoc test (n = 12, *p* < 0.05).

CP treatment can affect grains at different physiological levels. Germination kinetics of grains is important in plant development and is an indicator that the treatments cause changes in plant metabolism and development. In our study, CP treatment had a significant negative effect on the germination rate (G) of buckwheat grains, as all treated grains showed a lower G (Fig. 1a). The lowest G value after 10 days, measured in buckwheat grains subjected to the longest CP treatment time of 60 s, was only 25.8%, in comparison to the untreated grains value of 64.8%. The speed of germination (MR) was also influenced: CP-treated grains germinated much slower than untreated grains (Fig. 1b). However, there was no difference in MR between different CP treatments. Although the parameters of CP treatment were different, similar effects on buckwheat grain lowered G and MR were observed by Mravlje et al. (2021) [30]. Another study, conducted by Ivankov and colleagues [31], showed different responses to CP treatment of common buckwheat grains of two varieties. While ‘Vokiai’ variety did not exhibit any differences in G and MR in the *in vitro* study, the ‘Nojai’ variety showed a decrease in MR after 7 min CP treatment. The different types and parameters of plasma treatment used in previous studies make it difficult to compare results. However, common buckwheat grains are sensitive to CP treatment. A study that would describe improved germination parameters in *in vitro* settings has not yet been reported. Structurally, cotyledons of common buckwheat grain wind along the seed coat with only one layer of aleurone cells between the cotyledons and seed coat. Thus, the cotyledons are quite exposed to the environmental factors, as they are not protected by many layers of grain tissues and could be easily damaged in unfavourable conditions [32,33]. This may suggest that common buckwheat grains are very sensitive to one or more components of plasma treatments (or other environmental factors) and should be investigated further.

The G of both wheat grains (Primorka and Gorolka) was unchanged in CP treatments of 5 and 30 s (Fig. 1c–e). Only the longest treatment time of 60 s caused a significant decrease of G of both varieties. Primorka grains had slower MR only after the longest CP treatment (Fig. 1d), while Gorolka grains showed a slower MR after 30 s of treatment, with the slowest MR in grains treated for 60 s (Fig. 1f). G of control grains was already high (∼100%). Thus we could not see an impact of CP treatment in a possible increase in G, as reported by several published papers [[34], [35], [36], [37]]. For example, Ussenov and colleagues [11] reported an increase in G of CP-treated wheat grains for 5, 10 and 15 s with dielectric barrier discharge plasma. However, treatment of grains with CP for a long time caused a decrease in G, with the lowest G value (∼15%) reported after treatment for 60 s. Thus, when choosing the parameters for CP treatment of grains, it is necessary to obtain a dose-response curve, to determine optimal treatment conditions based on grain/plant response to plasma.

For G and MR, statistically significant differences between buckwheat and wheat variety Primorka, as well as buckwheat and wheat variety Gorolka, were found in all samples (control and CP-treated) as shown in Table 1. The different germination response of grains to CP treatment between common buckwheat grains and grains of two winter wheat cultivars is also variety and species-dependent, as was reported for several other species such as common and Tartary buckwheat [20] and wheat and oat grains [35]. When comparing G values of the Primorka and Gorolka wheat grains, the only statistically significant difference was noticed in grains treated with CP for 60 s. Here, G of Primorka grains (56.6%) was significantly lower compared to G of Gorolka grains (67.8%). This indicates that Gorolka grains are more resistant to the harmful effects of the selected CP treatments than Primorka grains. Two-way ANOVA found differences in the MR values of Primorka and Gorolka wheat grains, except for the 30 s CP treatment. Gorolka grains had higher MR values and germinated faster than Primorka, which accounted for all significant differences between the two varieties. Differences between the two varieties were also reported in previous studies [38]. Several authors have reported variety-dependent response to CP treatment in pea, rice and oilseed rape grains/seeds [15,19,39]. To better determine which components of plasma are important for grain response to CP treatment, an experimental setup with different grain mutants should be conducted to systematically approach the question, as was done on *Arabidopsis thaliana* mutants seeds [6]. The loss of moisture content during CP treatment [28,40], as well as a possible increase in surface temperature [28], could be important factors that trigger a response in plant metabolism. Thus, appropriate targets in the metabolism for the formation of mutant plants should be designed to further investigate if an increase in temperature and water loss from grains are important factors of plasma treatment that affect grain physiology.

**Table 1:** Table of *p*-values from two-way analysis of variance (two-way ANOVA), where the influence of variety/species of grain (buckwheat, wheat Primorka and wheat Gorolka) and time of CP treatment of grains (0, 5, 30 and 60 s) on germination (G), speed of germination (MR), α-amylase activity, phenol content and MDA equivalent were taken into consideration. Statistically significant p-values (*p* < 0.05) are marked in bold.

G				
	0	5	30	60
Buckwheat ˟ Primorka	**<0.001**	<0.001	<0.001	<0.001
Buckwheat ˟ Gorolka	**<0.001**	<0.001	<0.001	<0.001
Gorolka ˟ Primorka	0.193	0.845	0.608	<0.001
		MR		
	0	5	30	60
Buckwheat ˟ Primorka	**<0.001**	<0.001	<0.001	<0.001
Buckwheat ˟ Gorolka	**<0.001**	<0.001	<0.001	<0.001
Gorolka ˟ Primorka	**0.002**	0.016	0.286	<0.001
α-amylase activity				
	0	5	30	60
Buckwheat ˟ Primorka	<0.001	<0.001	**<0.001**	<0.001
Buckwheat ˟ Gorolka	<0.001	<0.001	**<0.001**	<0.001
Gorolka ˟ Primorka	0.319	<0.001	0.078	0.425
Phenol content				
	0	5	30	60
Buckwheat ˟ Primorka	<0.001	<0.001	**<0.001**	**<0.001**
Buckwheat ˟ Gorolka	<0.001	<0.001	**<0.001**	**<0.001**
Gorolka ˟ Primorka	0.006	0.019	0.093	**0.008**
MDA equivalent (lipid peroxidation)				
	0	5	30	60
Buckwheat ˟ Primorka	<0.001	<0.001	**<0.001**	**<0.001**
Buckwheat ˟ Gorolka	<0.001	<0.001	**<0.001**	**<0.001**
Gorolka ˟ Primorka	0.307	0.493	0.074	**<0.001**

**Fig. 2:**
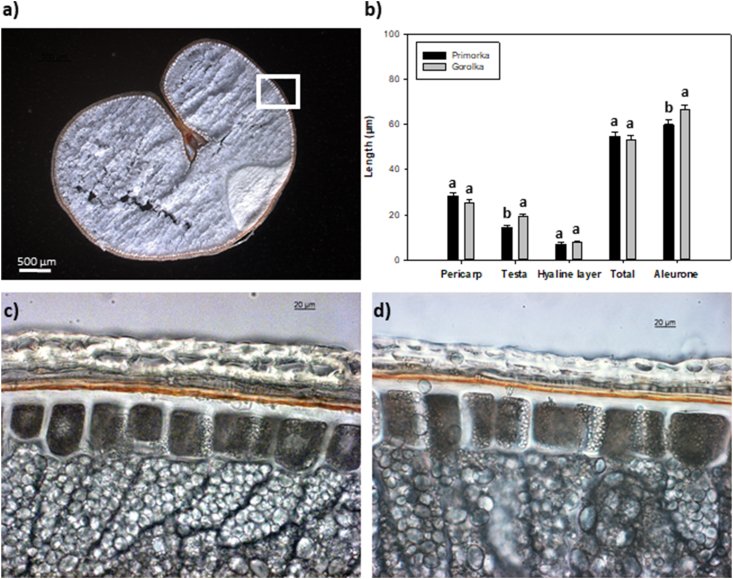
(**a**) Representative cross-section of wheat grain, white square marking the sampling region of grain coat measurements. (**b**) Average thickness and ±standard error of pericarp (P), testa (T), hyaline layer (H), the total thickness of grain coat (P + T + H) and the thickness of aleurone layer (A) in cross-section of Primorka and Gorolka wheat grains. Different letters (a–b) indicate statistically significant differences among treatments according to Holm-Sidak post-hoc test (n = 7, *p* < 0.05). A representative cross-section of Primorka (**c**) and Gorolka (**d**) grain under the light microscope.

The differences in response to CP treatment between Primorka and Gorolka grains described above might be due to structural differences, especially in the seed coat. To investigate structural differences between the two, measurements of pericarp layers were performed. In Fig. 2a, an example of wheat cross-section in shown. Under a light microscope, several layers of seed coat are visible: pericarp, testa and hyaline layer [41]. When comparing grain testa and aleurone thickness, statistically significant differences in the thickness of the layers were observed (Fig. 2b). Gorolka grains have a thicker testa layer compared to Primorka grains. The function of the testa is to protect the plant embryo against different environmental conditions during grain storage. Representative cross-sections of wheat Primorka and Gorolka are shown in Fig. 2c and d.

**Fig. 3:**
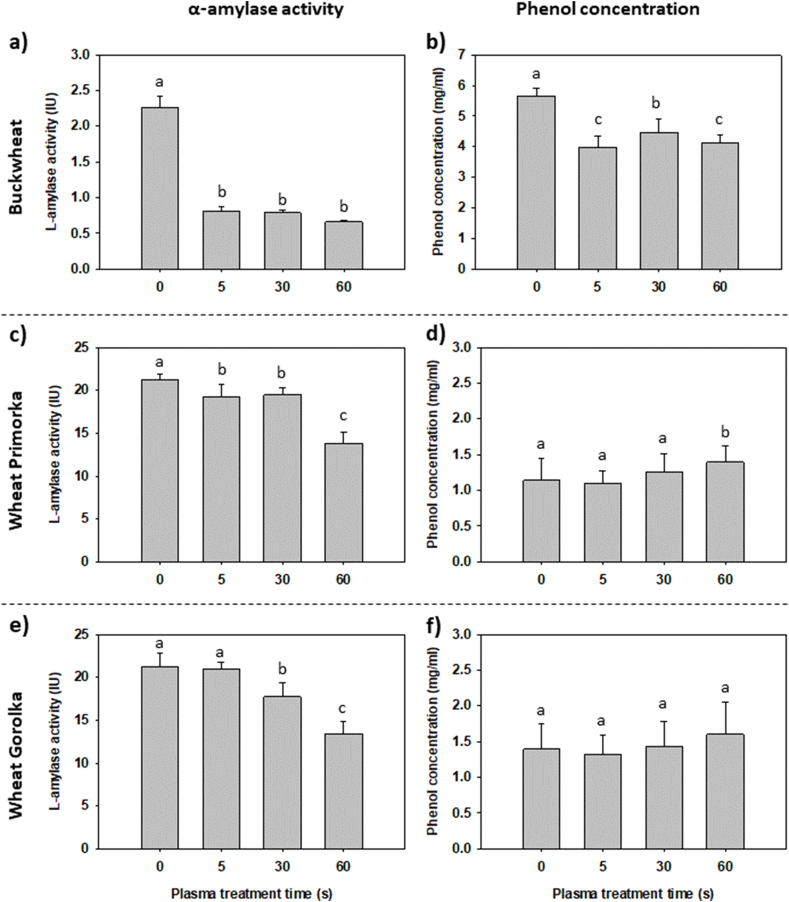
(a, c, e) Activity of α–amylase enzyme and (b, d, f) phenol concentration on grain pericarp of untreated (0 s) grains and CP-treated grains for 5, 30 or 60 s was measured. Displayed values are the mean ± SE of three replications. Different letters (a–c) indicate statistically significant differences among treatments according to Holm-Sidak post-hoc test (n = 9, *p* < 0.05).

The activity of α-amylase enzyme is tightly linked to germination parameters. During germination, α-amylase participates in releasing simple carbohydrates from large molecules of starch in the grain endosperm. All CP treatments of Buckwheat grains caused a significant decrease in α-amylase activity compared to control samples (Fig. 3a). However, no differences were measured between CP treatments. Lower enzyme activity could be correlated with lower G and MR of CP-treated buckwheat grains. This effect shows a response of grains to CP treatment, with slowing or inhibiting the metabolism involved in germination. In a study including CP treatment of common buckwheat grains for the purpose to biofortify sprouts with zinc, no statistically significant changes in α-amylase activity were found [42]. However, the difference in the results might also be due to the lower input power used, as well as the different treatment time intervals.

The observed gradual decrease in α-amylase enzyme activity with longer CP treatment times for wheat grains (Fig. 3c, e) confirms that plant metabolism was affected by CP, and the germination process was slowed, resulting in a lower final germination rate. A significant increase in α-amylase activity of wheat grains was reported in another study, where G after CP treatment also increased [11]. Similar results were found in rice grains and mung bean seeds, indicating a close connection between changes in α-amylase activity and germination parameters. The response to CP treatment depended on the species/variety's susceptibility to adjust to environmental factors [12,43].

When comparing buckwheat and wheat variety Primorka, as well as buckwheat and wheat variety Gorolka, statistically significant differences were noticed between species (Table 1). Interestingly, hardly any significant difference in α-amylase activity between Primorka and Gorolka wheat varieties was detected even though there were differences in germination response to CP treatment. This indicates that other signalling pathways, apart from α-amylase activity, were influenced by CP treatment, resulting in a difference between germination response and enzyme activity. Each variety responds and compensates the stressors from CP treatment differently, which results in different germination patterns and causes changes in grain metabolism.

### 3.2 Total phenolic content in grain pericarp and lipid peroxidation of grains

CP is a surface technique, and its direct effect on a treated sample is generally considered limited to a depth of a few nm to 1 mm. However, the penetration depth of plasma components, such as VUV/UV radiation and reactive species, depends on the porosity of the treated material, chemical composition, as well as the characteristics of the CP used for the treatment [2,3]. The predicted penetration depth of reactive species generated in plasma treatment of seeds for Mung beans, for example, is around 3 nm [3]. As CP treatment affects the surface chemistry of grains, we examined the influence of CP treatment on the phenol content in grain pericarp. CP treatment of buckwheat grains significantly reduced the phenol content (Fig. 3b). On the other hand, almost no differences were found between CP-treated wheat grain pericarps (Fig. 3d, f). It is speculated that phenols in buckwheat play an important role in mitigating abiotic stresses, such as temperature extremes and UV-B radiation [44]. Plasma reactive species may alter important phenols for buckwheat grain germination upon oxidation, leading to changes in the germination parameters. Phenols can exist in tissues as free or bound forms. In buckwheat, the free phenol content in hulls constitutes 80–95% of the total phenol content, according to Li et al. [45]. In contrast, phenols in wheat are mainly present in a bound form [[45], [46], [47]]. The bound form of phenolic compounds in wheat grains may be more resistant to cold plasma oxidation than the free form in buckwheat pericarp, where the molecules are more exposed to the reactive oxygen species of plasma discharge.

**Fig. 4:**
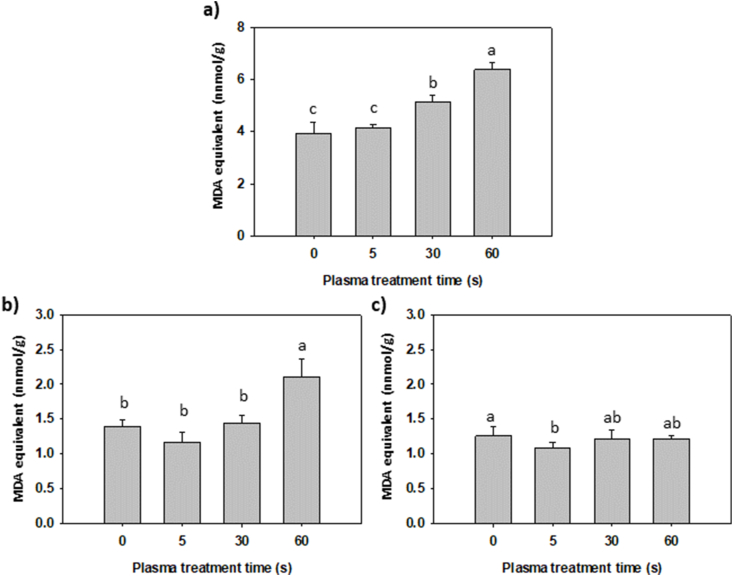
MDA equivalent concentrations in buckwheat (**a**), wheat Primorka (**b**) and wheat Gorolka (**c**) grains treated with CP for 0, 5, 30 or 60 s. Displayed values are the mean ± SE of three replications. Different letters (a–c) indicate statistically significant differences among treatments according to Holm-Sidak post-hoc test (n = 6, *p* < 0.05).

Lipid peroxidation is usually detected through a biomarker called malondialdehyde (MDA). The process is activated when reactive oxygen species “attack” lipids in cells and cause oxidation of the polyunsaturated fatty acids [48,49]. As plasma contains ROS and could cause a reduction in moisture content in grains because of low-pressure and higher surface temperatures, we also measured MDA content in grains after CP treatment. In buckwheat grains, CP treatment for 5 s did not increase the MDA content (Fig. 4a). However, longer treatment times (30 s and 60 s) exhibited higher content of MDA compared to untreated grains. In Primorka wheat grains, MDA content increased only in grains treated with CP for 60 s (Fig. 4b). On the other hand, grains of Gorolka wheat did not exhibit any significant changes in MDA content after CP treatment (Fig. 4c). In a different study, the CP treatment of oilseed rape seeds led to a decrease in MDA content, indicating that the CP treatment reduced MDA production [15]. However, an increase in MDA content after CP treatment was reported in wheat and rice grains [16,17]. Guo and colleagues also observed an outward diffusion of certain intracellular components and an increase in the electrical conductivity of the grains, indicating that the CP treatment affected the cell membrane permeability of rice grains [17].

Two-way ANOVA showed statistically significant differences in MDA content between buckwheat and Primorka grains, as well as buckwheat and Gorolka grains for all treatments. However, when comparing Primorka and Gorolka grains, it was observed that 60 s CP treatment exhibited statistically significant differences between the two varieties (Table 1). MDA equivalent measured in 60 s CP-treated Gorolka grains (1.21 nmol/g) was much lower than Primorka grains (2.10 nmol/g), implying that lipid peroxidation in Gorolka grains was not activated. This could be due to the greater resistance of Gorolka wheat to temperature stresses. In a study conducted by Ristic et al. [50], it was found that Gorolka was one of only two wheat varieties that displayed immunity to heat stress, as evidenced by its ability to maintain its chlorophyll content and the heat stability of its thylakoid membranes, without experiencing any significant changes.

CP treatment of grains also causes an increase in the temperature of the grain surface. However, the temperature depends on the plasma parameters, as well as the time of the treatment. Rough estimates of the surface temperature of grains during CP treatment of wheat grains in similar conditions were measured. The temperature steadily rose with longer CP treatment time. At 5 s CP treatment, the estimated temperature of the grain surface was 32 °C, with the ambient temperature at 25.5 °C at the start of the experiment. After 30 s of CP treatment, the temperature rose to 62 °C and after 60 s to 85 °C (unpublished results). Thus, the reduction in the germination of grains, as well as the rise in MDA content after 60 s due to the rise in temperature is highly probable. The process of lipid peroxidation is highly dependent on the moisture content of grains, with both low and high moisture levels capable of activating the process [51]. In the case of cold plasma treatment, the low-pressure can cause a slight loss of moisture in the grains. Furthermore, the increased temperature and the presence of reactive oxygen species in the plasma during CP treatment could also activate the process of lipid peroxidation, especially during longer treatment times.

### 3.3 Fourier transform infrared (FTIR) analysis

CP treatment is known to change the surface chemistry of polymers and seeds/grains [7,52]. Plasma species react with the grain surface molecules. In case of oxygen plasma treatment, mostly oxidation of surface molecules occurs. Buckwheat and wheat pericarps show more hydrophobic properties and have a waxy layer of lipids. The hypothesis that the CP treatment caused the oxidation of lipids on the grain surface of buckwheat and wheat grains was investigated with FTIR.

**Fig. 5:**
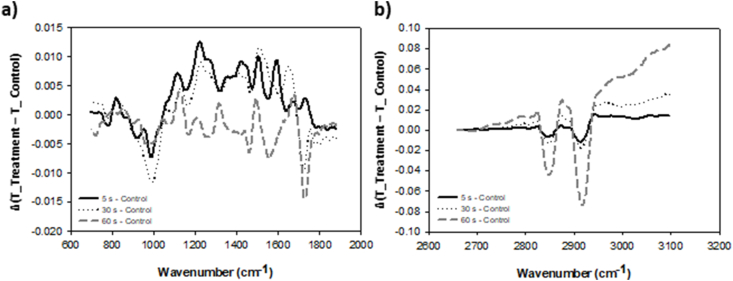
FTIR spectrum of buckwheat pericarp in **(a)** fingerprint and **(b)** lipid region of the spectrum, where transmittance (T) of untreated grains (Control) was deducted from plasma treated (5 s, 30 s or 60 s) samples.

In all CP-treated samples of buckwheat, the signal in the frequency range 2852–2917 cm^−1^ decreased with longer CP treatment times in comparison to untreated (control) samples (Fig. 5b). This range is associated with strong C–H stretching vibrations of alkanes and/or lipid molecules, indicators of CP treatment oxidation on lipid molecules in the grain pericarp [28,53,54]. Significant changes are also notable around 1730 cm^−1^ peak (Fig. 5a), which is associated with strong aldehyde and/or α,β-unsaturated esters C=O stretching. These changes might also be related to the oxidation of lipid molecules by CP treatment.

**Fig. 6:**
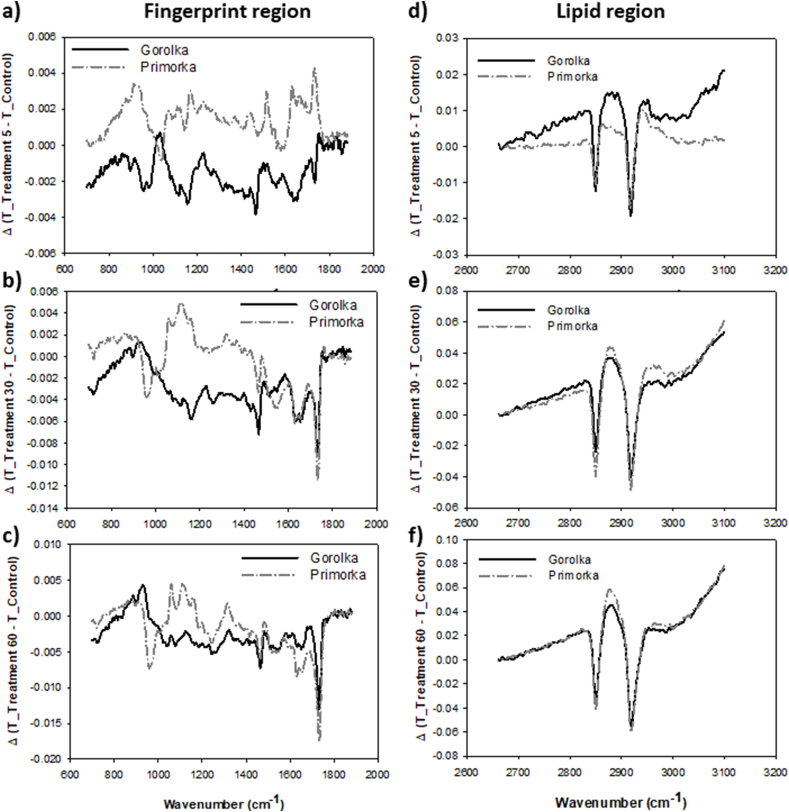
FTIR spectra in **(a**–**c)** fingerprint and **(e**–**f)** lipid area of wheat Primorka and wheat Gorolka grains untreated or treated with CP for 5, 30 and 60 s. The measured absorbance of untreated control grain pericarp was deducted from the measured absorbance of each plasma treatment.

In the fingerprint region, especially around peaks with a wavenumber of approximately 1730 cm^−1^ (Fig. 6a–c) and in the lipid region, with peaks at 2850 and 2920 cm^−1^(Fig. 6e–f), differences between Primorka and Gorolka wheat pericarp were detected, as well as the differences between different CP treatments. In the lipid region, wheat Gorolka exhibited less changes than Primorka wheat, which may also be in connection with lower MDA equivalent measured in Gorolka grains. A greater change around 1730 cm^−1^ peak, which signifies C=O stretching, in Primorka pericarp and 60 s CP treatment (Fig. 6c) might also be connected to higher lipid peroxidation measured in Primorka grains than in Gorolka grains.

**Fig. 7:**
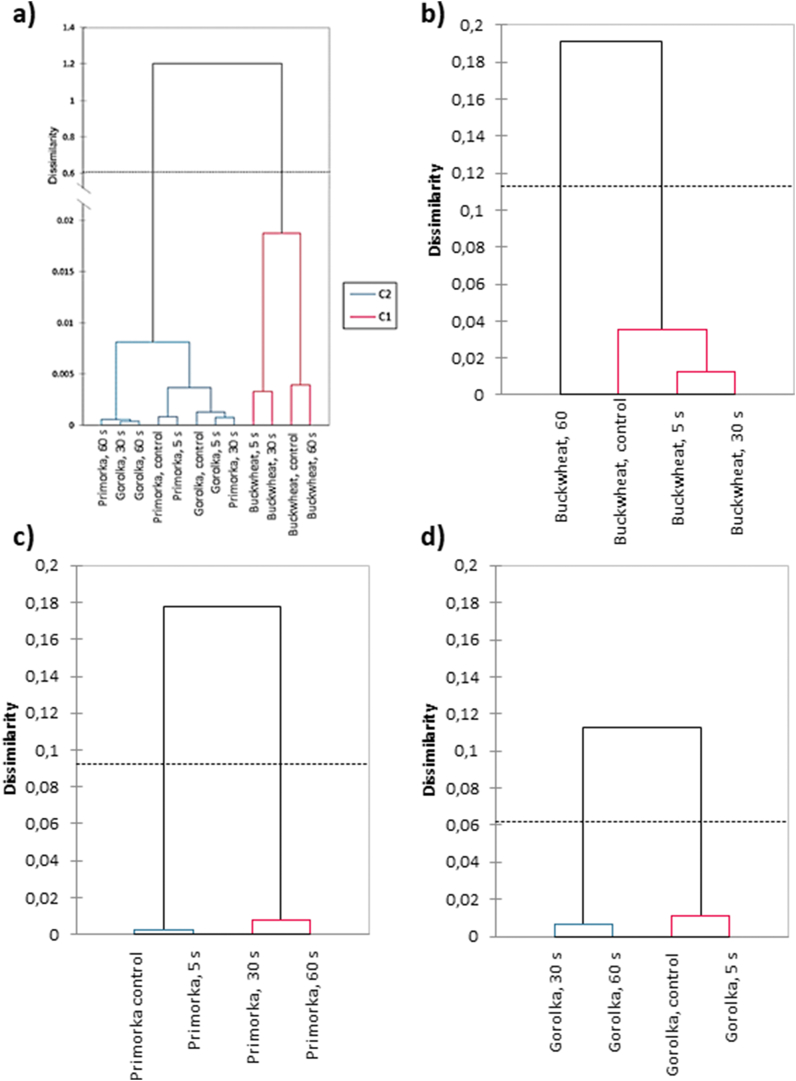
Dendrograms of average FTIR spectra of untreated or CP-treated buckwheat, wheat Primorka and wheat Gorolka grains for 5, 30 or 60 s. Dendrograms represent the dissimilarities between **(a)** buckwheat and both wheat varieties, **(b)** dissimilarities between different CP treatments and untreated buckwheat grains, **(c)** dissimilarities between different CP treatments and untreated wheat Primorka grains and **(d)** dissimilarities between different CP treatments and untreated wheat Gorolka grains.

When comparing similarities in FTIR spectral profiles of CP-treated grains of buckwheat, wheat Primorka and wheat Gorolka, it is evident that profiles of both wheat varieties are most similar (Fig. 7a). The largest dissimilarity is seen between buckwheat and both wheat varieties, as expected. Control and CP-treated grains for 5 s of both Primorka and Gorolka grains exhibit similar infrared spectra and are dissimilar to the spectra of CP-treated grains for 30 s and 60 s, where with longer exposure to CP treatment, more chemical functionalization of pericarp surface is seen (Fig. 7c and d). On the other hand, buckwheat grains treated with CP for 5 and 30 s exhibit similar spectral profiles, but differ slightly from the untreated grains (Fig. 7b). The longest CP treatment time (60 s) caused changes in the chemical structure of the pericarp, evident in a larger dissimilarity to other samples.

## 4 Conclusion

The CP treatment has been shown to affect different species and varieties in different ways. Buckwheat grains, for example, appear more susceptible to the negative effects of CP treatment than grains of winter wheat. This susceptibility is immediately noticeable in delayed germination and a decrease in the germination rate. This could be due to differences in the structure of grain tissues as well as chemical differences, such as a higher concentration of free phenolic compounds in the grain pericarp, which is the most exposed part of the grain during plasma treatment. The wheat embryo inside the grain is structurally more protected than the buckwheat grains, where cotyledons are aligned close to the grain pericarp and surface. In contrast, wheat grains were generally more resistant to the negative effects of CP treatment, which only became evident after prolonged exposure. However, differences in response to CP treatment have been observed between two wheat varieties, Primorka and Gorolka. These differences may be attributed to subtle differences in the chemical structure of the pericarp, the thickness of the grain testa, and possible differences in genetics, which could govern the metabolic response to CP treatment. The Gorolka wheat variety was found more resistant to the stress caused by CP treatment, suggesting that it may be a useful candidate for further investigation and development of CP treatment as a stressor for wheat grains. In the end, these findings highlight the need for further research to elucidate the mechanisms underlying the variable response of different grain species and varieties to CP treatment, which could ultimately lead to the development of more effective and targeted treatments for specific grain varieties.

## Funding

The work was funded by the 10.13039/100002599Public agency for scientific research and innovation activity of the Republic of Slovenia (10.13039/100007262ARIS) through program groups [P2-0082, P1-0212, P4-0072, and P1-0112], ARIS young research grant [PS], ARIS project [ J1-3014] and project supported by ARIS and 10.13039/501100003993Ministry of agriculture, forestry and food [CRP V4-2001].

## Author contribution statement

Pia Starič;, Katarina Vogel-Mikuš: Conceived and designed the experiments; Performed the experiments; Analyzed and interpreted the data; Wrote the paper. </p>

Aleš Kolmanič, Ita Junkar: Analyzed and interpreted the data; Contributed reagents, materials, analysis tools or data; Wrote the paper. </p>

## Data availability statement

Data will be made available on request.

## Declaration of competing interest

The authors declare that they have no known competing financial interests or personal relationships that could have appeared to influence the work reported in this paper.
